# Excess Body Weight and Cancer Risk in Patients with Type 2 Diabetes Who Were Registered in Swedish National Diabetes Register – Register-Based Cohort Study in Sweden

**DOI:** 10.1371/journal.pone.0105868

**Published:** 2014-09-08

**Authors:** Junmei Miao Jonasson, Jan Cederholm, Soffia Gudbjornsdottir

**Affiliations:** 1 Social Medicine, Department of Public Health and Community Medicine, Sahlgrenska Academy, University of Gothenburg, Gothenburg, Sweden; 2 Nordic School of Public Health NHV, Gothenburg, Sweden; 3 Department of Public Health and Caring Sciences/Family Medicine and Preventive Medicine, Uppsala University, Uppsala, Sweden; 4 Registercenter, VGR, Gothenburg, Sweden; 5 Department of Medicine, Sahlgrenska Academy, University of Gothenburg, Gothenburg, Sweden; University of Bari Aldo Moro, Italy

## Abstract

**Aim:**

To assess the association between excess body weight and cancer risk in patients with type 2 diabetes (T2D) who were registered in the Swedish National Diabetes Register (NDR).

**Methods:**

This is a cohort study based on 25,268 patients with T2D and baseline BMI≥18.5 kg/m^2^ from NDR 1997–1999. Subjects were grouped according to BMI into normal weight (18.5 to 24.9), overweight (25 to 29.9) or obesity (30 or more). All subjects were followed until the first occurrence of cancer, or death, or the end of follow-up (December 31, 2009). Adjusted hazard ratios (HRs) and 95% confidence interval (CI) for cancer risks were estimated by Cox regression.

**Results:**

In men with T2D, overweight was associated with increased risks of all cancer [1.13 (1.02–1.27)], gastrointestinal cancer [1.34 (1.07–1.72)] and colorectal cancer [1.59 (1.18–2.13)]; obesity was related to higher risks of all cancer [1.17 (1.04–1.33)], gastrointestinal cancer [1.40 (1.08–1.82)] and colorectal cancer [1.62 (1.17–2.24)]. In women with T2D, obesity was associated with increased risk of all cancer [1.30 (1.12–1.51)], gastrointestinal cancer [1.40 (1.03–1.91)] and postmenopausal breast cancer [1.39 (1.00–1.91)].

**Conclusions:**

Excess body weight was associated with increased risks of all cancer, gastrointestinal cancer and colorectal cancer in men with T2D. Obesity was related with elevated risks of all cancer, gestational cancer and postmenopausal breast cancer in women with T2D.

## Introduction

Patients with type 2 diabetes (T2D) have been shown to be associated with increased risk of cancer compared to non-diabetic subjects [1]. The underlying mechanisms remain unclear. Excess body weight, i.e., overweight or obesity, and hyperglycemia have been speculated to be possible risk factors for increased risk of cancer occurrence in patients with T2D [1]. Our recent study did not find any association between hyperglycemia and cancer risks in patients with T2D [2].

Limited information is available on the association between excess body weight and cancer risk in patients with T2D. In general populations, overweight and obesity have been reported to be associated with increased risk for all cancer, gastrointestinal cancer, colorectal cancer and postmenopausal breast cancer [3]–[7]. A positive association between excess body weight and prostate cancer was reported in some studies [4], [5], but not in others [3]. Until now, only one study has investigated this association in patients with T2D and found that obesity was inversely related to total cancer incidence [8]. However, the authors could not rule out reversed causation due to short follow-up period. To our best knowledge, there are no previous reports in the literature on the association between excess body weight and risk of cancer of specific sites among patients with T2D.

This register-based cohort study aims to investigate the association between excess body weight and risks of all cancer and specific types of cancer like gastrointestinal cancer, colorectal cancer, breast cancer and prostate cancer, in patients with T2D who were registered in the Swedish National Diabetes Register (NDR) during the period from 1997 to 1999.

## Materials and Methods

This is a cohort study based on register-linkage of Swedish NDR, the Swedish Cancer Register and the Swedish Causes of Death Register. More details about these registers have been previously described [9]. The study cohort included patients with T2D who were registered in NDR during 1997 to 1999. All included patients have agreed by informed consent to be registered in the NDR before inclusion. Cohort members were followed by linkage to the Cancer Register and the Causes of Death Register [2].The Regional Ethics Review Board at the University of Gothenburg approved this study.

The study cohort consisted of 25,268 patients with type 2 diabetes and BMI≥18.5 kg/m^2^, with baseline information available for all variables used in the study during the 1997–1999 period. The age range was 30–90 years. We did not include patients with BMI<18.5 kg/m^2^ since the development of undiagnosed cancer might cause patients to be underweight. Further exclusion criteria included diagnosis of cancer of study interest, or death before the start of follow-up, as obtained through linkage to the Cancer Register and the Causes of Death Register. Cohort members were followed from the first day of the year after the baseline clinical examinations in 1997–1999 until the first diagnosis of an outcome, or death, or the censor date December 31, 2009. Study outcomes were the first diagnosis of any malignant cancer, or the first diagnosis of gastrointestinal cancer, colorectal cancer, prostate cancer and breast cancer during follow-up.

Baseline BMI (kg/m^2^), calculated as weight (kg) divided by height (m) squared, was categorized into: 18.5 to 24.9 (normal weight), 25 to 29.9 (overweight), 30 kg/m^2^ or more (obesity). BMI was also measured over time as an updated mean of annual measurements, using last observation carried forward (LOCF) in case of missing data. Missing data on BMI during follow-up were present among 5–10% of the patients. To further check the effect of reversed causation, i.e., undiagnosed cancer might lead to the change of BMI during the two years before the end of follow-up, we did not count the BMI values measured during these two years.

### Ethics

The data linking of national registers required for this study was approved by the Regional Ethics Review Board at the University of Gothenburg. All data analyzed were anonymous; therefore, informed consent for each individual was neither necessary according to Swedish legislation act 2003∶460 concerning research on humans, nor is it possible when data is anonymous.

### Statistical methods

The Cox proportional hazard regression was used to estimate hazard ratios (HR) with 95% confidence interval (CI) for risk of cancer by BMI as predictor. Baseline BMI was firstly treated as a continuous variable with increase per 5 units, and secondly also as a categorical variable by comparing overweight or obese groups to a normal weight group. Follow-up time was used as time scale. Adjustment was made for potential confounding factors: age, gender, diabetes duration, HbA1c, smoking and insulin treatment. The proportional hazard assumption was evaluated using graphs of scaled Schoenfeld residuals. A violation of the proportional hazards assumption was detected for age when analysing all cancer, or prostate cancer, as outcomes, and this was corrected by using quartiles of age as strata in these Cox models.

Updated mean BMI was also analyzed, applied as a strictly time dependent variable in the Cox model, and allowing to use the most recent in-study level at each specific point of time in the modeling process. In case of an event during follow-up, BMI was the value of the year before this event, otherwise BMI was the value of the censor year.

We did sensitive analysis by excluding subjects with cancer of interest in the first two years after the start of the observation period for all cancer, prostate cancer in men and breast cancer in women. All statistical analyses were performed using SAS 9.3 (SAS Institute, US).

## Results

A total 25,268 T2D patients were divided into three groups according to baseline BMI. The median follow-up was 8.6±3.5 years. Table 1 presents baseline clinical characteristics in the three groups, where 23.6% had normal weight, 43.3% were overweight, and 33.1% were obese. Mean age, diabetes duration and Hba1c differed slightly between the groups, while normal weight patients were more frequently insulin treated (Table 1).

**Table 1:** Baseline characteristics in all patients with type 2 diabetes, and by different categories defined according to baseline BMI.

	Baseline BMI categories (kg/m^2^)			
	Normal weight	Overweight	Obese	P
	BMI 18.5–24.9	BMI 25–29.9	BMI≥30	value
Numbers	5,969	10,949	8,350	
Age, years	67.9±12.1	66.7±11.1	64.0±10.9	<0.001
Diabetes duration, years	10.3±8.5	9.1±7.5	8.0±6.8	<0.001
HbA1c, %	7.6±1.4	7.6±1.3	7.8±1.4	<0.001
Male gender	53.8	61.7	50.4	<0.001
Smoker	15.3	12.2	12.5	<0.001
Insulin treatment	47.4	40.9	38.5	<0.001

Data given as means ± SD or frequencies (%). P value for trend across groups (ANOVA).

In all T2D patients, the adjusted hazard ratios for every 5 units increase in baseline BMI were 1.08 (1.04–1.12), for all cancer 1.08 (1.01–1.17) for gastrointestinal cancer and 1.11 (1.01–1.21) for colorectal cancer. Compared with normal weight patients, increased hazard ratios for the above mentioned cancers were observed in both overweight and obese patients (Table 2).

**Table 2:** Hazard ratios (HR) with 95% confidence intervals (CI) for all cancer and specific types of cancer, comparing overweight or obese patients with normal weight patients as reference, classified based on baseline BMI values, given for all patients with type 2 diabetes and separately in men and women.

	Per 5 units	Baseline BMI categories (kg/m^2^)					
	increase	Normal weight		Overweight		Obese	
	in BMI	BMI 18.5–24.9		BMI 25–29.9		BMI≥30	
	HR (95% CI)*	Cases	HR	Cases	HR (95% CI)*	Cases	HR (95% CI)*
**All patients**							
All cancer	1.08 (1.04–1.12)	714	1.0	1554	1.13 (1.03–1.23)	1150	1.22 (1.11–1.34)
Gastrointestinal cancer	1.08 (1.01–1.17)	159	1.0	385	1.29 (1.07–1.55)	279	1.41 (1.15–1.72)
Colorectal cancer	1.11 (1.01–1.21)	109	1.0	276	1.35 (1.08–1.69)	206	1.52 (1.20–1.93)
**Men**							
All cancer	1.05 (1.00–1.11)	449	1.0	1076	1.13 (1.02–1.27)	629	1.17 (1.04–1.33)
Gastrointestinal cancer	1.06 (0.95–1.18)	96	1.0	267	1.34 (1.07–1.72)	149	1.40 (1.08–1.82)
Colorectal cancer	1.10 (0.96–1.25)	59	1.0	192	1.59 (1.18–2.13)	106	1.62 (1.17–2.24)
Prostate cancer	1.00 (0.92–1.10)	160	1.0	384	1.13 (0.94–1.36)	192	1.01 (0.81–1.25)
**Women**							
All cancer	1.10 (1.05–1.16)	265	1.0	478	1.13 (0.97–1.32)	521	1.30 (1.12–1.51)
Gastrointestinal cancer	1.10 (0.99–1.22)	63	1.0	118	1.16 (0.86–1.58)	130	1.40 (1.03–1.91)
Colorectal cancer	1.11 (0.99–1.26)	50	1.0	84	1.05 (0.74–1.49)	100	1.39 (0.98–1.96)
Breast cancer (all)	1.14 (1.03–1.26)	67	1.0	100	0.95 (0.69–1.29)	140	1.30 (0.97–1.75)
Postmenopausal							
breast cancer	1.19 (1.07–1.33)	55	1.0	88	0.96 (0.68–1.34)	120	1.39 (1.00–1.91)

*Adjusted for age, HbA1c, smoking, diabetes duration, diabetes medication (insulin yes or no).

First incident of all cancer was defined by means of ICD-10 codes (C00-C97, D00-D09, D37-D48) and the following specific types of cancer were also investigated: the first incident of gastrointestinal cancer (ICD-10 code C15–C25), colorectal cancer (C18–C21), breast cancer in all women (C50) as well as in women over 55 years at baseline (C50), and prostate cancer in men (C61). For cancer of specific sites, we included only tumors that were histopathologically classified as adenocarcinoma (WHO/HS/CANC/24.1 histology code 096).

Among men with T2D, the adjusted hazard ratios for every 5 units increase in baseline BMI were 1.05 (1.00–1.11) for all cancer (Table 2). Compared to normal weight men, both overweight and obese men had increased risk of all cancer, gastrointestinal cancer and colorectal cancer. No significant association was observed between obesity and prostate cancer. Among women with T2D, every 5 units increase in BMI was associated with an increased risk of all cancer, all breast cancer and postmenopausal breast cancer. Obesity was significantly associated with increased risk of all cancer, 1.30 (1.12–1.51), all gastrointestinal cancer, 1.40 (1.03–1.91), and postmenopausal breast cancer, 1.39 (1.00–1.91).

Table 3 shows an analysis using updated mean BMI until two years before the end of follow-up. Increased all cancer risk was observed for every 5 units increase in updated mean BMI in total, men and women, respectively. Comparing to normal weight, elevated all cancer risk was observed in overweight or obese groups.

**Table 3:** Hazard ratios (HR) with 95% confidence intervals (CI) for all cancer, in overweight or obese patients compared with normal weight patients as reference, classified based on updated mean BMI values, given in all patients with type 2 diabetes and by gender.

	Per 5 units	updated mean BMI categories (kg/m^2^)		
	increase	Normal weight	Overweight	Obese
	in BMI	BMI 18.5–24.9	BMI 25–29.9	BMI≥30
	HR (95% CI)*	HR	HR (95% CI)*	HR (95% CI)*
All patients	1.09 (1.05–1.13)	1.0	1.13 (1.03–1.24)	1.22 (1.11–1.35)
Men	1.06 (1.00–1.12)	1.0	1.13 (1.01–1.26)	1.19 (1.05–1.35)
Women	1.12 (1.06–1.18)	1.0	1.17 (1.01–1.36)	1.29 (1.11–1.50)

*Adjusted for age, HbA1c, smoking, diabetes duration, diabetes medication (insulin yes or no).

We did additional sensitivity analyses by adjusting with three different Cox models: 1) controlling for gender and quartiles of age as strata; 2) also controlling for the diabetes parameters duration and HbA1c; 3) additionally controlling also for insulin treatment. Both overweight and obesity were significantly associated with increased risk of all cancer in these three models (Table 4).

**Table 4:** Hazard ratios (HR) with 95% confidence intervals (CI) for all cancer in overweight and obese patients with type 2 diabetes, compared to patients with normal weight, using several models for adjustment with covariates.

	Per 5 units	Baseline BMI categories (kg/m^2^)		
	increase	Normal weight	Overweight	Obese
	in BMI	BMI 18.5–24.9	BMI 25–29.9	BMI≥30
Adjustment models	HR (95% CI)	HR	HR (95% CI)	HR (95% CI)
Model 1	1.08 (1.04–1.12)	1.0	1.12 (1.03–1.23)	1.22 (1.11–1.34)
Model 2	1.01 (0.98–1.03)	1.0	1.14 (1.04–1.25)	1.16 (1.06–1.28)
Model 3	1.05 (1.01–1.08)	1.0	1.15 (1.05–1.25)	1.18 (1.07–1.29)

Model 1: adjustment for gender and also stratification by quartiles of age in order to avoid violation by age of the proportional hazards assumption at Cox regression analysing all cancer.

Model 2: Diabetes duration and HbA1c added as covariates, except for gender and stratification by age quartiles.

Model 3: Insulin treatment also added as covariate, except for gender, duration, HbA1c, and stratification by age quartiles.

To avoid the risk of having subjects with undiagnosed cancer in our cohort, we conducted sensitivity analysis by excluding subjects who diagnosed cancer of interest within the first two years after start follow-up. For the sensitivity analysis of all cancer in all participants, prostate cancer in male participants and breast cancer in female participants, the results were similar.

Hazard ratios for all included covariates in the Cox regression analyses are presented in Tables S1 and S2.

## Discussion

We found that excess body weight was related to higher risks of all cancer, gastrointestinal cancer and colorectal cancer in patients with T2D who were registered in Swedish NDR 1997–1999. These increased risks were also observed separately in men. Among women, obesity was associated with increased risks of all cancer, all gastrointestinal cancer and postmenopausal breast cancer.

The mechanism by which excess body weight increases the risk of cancer is not fully understood. The insulin and insulin-like growth factor (IGF) axis, sex steroids and adiponectin have been considered potential explanations. Excess body weight is related to higher level of insulin and insulin-like growth factor I (IGF-1) which promote certain types of tumor cell proliferation and growth. There was no important link between IGF-1 levels and postmenopausal breast cancer [10]. A strong association between postmenopausal breast cancer risk and estrogens and androgens levels has been well confirmed [10]. Adiponectin, negatively correlated with BMI, is inversely associated with occurrence of several types of cancer [11], such as breast cancer and colon cancer [12].

The effects of excess body weight on cancer risk found in patients with T2D are consistent with studies based on general population. Compared to normal weight, obesity was associated with elevated risks of gastrointestinal cancer, colorectal cancer, postmenopausal breast cancer in a Swedish cohort study [3], in the Million Women Study in UK [13], and in a meta-analysis of epidemiological studies from Europe [6]. Overweight has also been related to increased risks of postmenopausal breast cancer and colon cancer [6]. Some studies in the general population [4], [6], but not all [3], showed a positive association between excess body weight and risk of prostate cancer.

We found that excess body weight was associated with an increased risk of colorectal cancer in men, but not in women, with T2D. One explanation could be that 74% of women in our study cohort were aged over 55 years, among which no association between obesity and colorectal cancer was found in a previous study [14]. A similar gender difference was reported in a meta-analysis of prospective studies in general population [7]. Another explanation could be due to hormone differences between men and women. Furthermore, a difference in the distribution of body fat is often seen between men and women. Compared to women, men have more central adiposity and more visceral adipose tissue [15], which might be important for the risk of colon cancer [16].

Our finding of the positive association between obesity and postmenopausal breast cancer is consistent with reports among women in general population [17], [18]. In obese postmenopausal women an increased risk of breast cancer might be due to factors related to their obesity, such as increased estrogen levels [19] and a decreased adiponectin level [20], which may elevate breast cancer cell proliferation.

Until now, there has been only one report on the association between obesity and cancer risks in patients with T2D. It was based on 26,742 patients with T2D in Germany with a median follow-up of 3.5 years. BMI≥30 kg/m^2^ was associated with decreased total cancer incidence [8]. The authors could not rule out reversed causality as possible explanation, due to the relatively short follow-up period. Comparatively, this study had median 8.6 years of follow-up, and we observed increased cancer risks with overweight or obesity when using baseline BMI as predictor. To further check the effect of reversed causation, we also measured the effect of updated mean BMI until two years before the end of follow-up, and similar results of increased cancer risks were observed. Thus, it is unlikely that the observed associations in this study were due to reversed causality.

The main strengths of our study are cohort design, a large sample size based on high quality Swedish registers, a long follow-up period, complete information on baseline variables and cancer outcomes, and the possibility to adjust for relevant potential confounding factors. Since all study data were measured and recorded by trained health professionals, there was no concern of recall bias. Since our prospective study had well-documented information on body weight and height values, and also on diagnosis of diabetes and on cancer incidence, this made it possible to determine the temporal sequence of the causal relationship, if any.

Our study has some limitations. Some information was not available in our register, we had no data on sum of comorbidities, on specific oral hypoglycaemic drugs, or alcohol consumption. Data on insulin treatment were reported at baseline of the study, used as covariate in the Cox regressions. Patients with type 2 diabetes with such a degree of a deranged glucose metabolism that they had need for insulin at baseline should reasonably be in need for insulin also during the study period. Certain specific types of cancer, such as endometrial, pancreatic or liver cancer, could not be investigated in our study due to the small numbers of outcomes.

*In conclusion*, excess body weight was associated with increased risks of all cancer, gastrointestinal cancer and colorectal cancer in men with T2D, and obesity was associated with increased risks of all cancer, gastrointestinal cancer and postmenopausal breast cancer in women with T2D.

## Supporting Information

Table S1 **Hazard ratios (HR) with 95% confidence intervals (CI) for all cancer and specific types of cancer, with BMI and all covariates given in the table, in patients with type 2 diabetes using the model with BMI per 5 units increase as main exposure.**
(DOCX)

Table S2 **Hazard ratios (HR) with 95% confidence intervals (CI) for all cancer and specific types of cancer, with BMI and all covariates given in the table, in patients with type 2 diabetes using the model with categorized baseline BMI as main exposure.**
(DOCX)
