# Supplementary material for: Excess Body Weight and Cancer Risk in Patients with Type 2 Diabetes Who Were Registered in Swedish National Diabetes Register – Register-Based Cohort Study in Sweden
Source: PLoS One. 2014 Sep 8;9(9):e105868. doi: 10.1371/journal.pone.0105868 (PMC4157768; doi:10.1371/journal.pone.0105868)
Supplement: Table S1 — Hazard ratios (HR) with 95% confidence intervals (CI) for all cancer and specific types of cancer, with BMI and all covariates given in the table, in patients with type 2 diabetes using the model with BMI per 5 units increase as main exposure. (DOCX) [file pone.0105868.s001.docx]

**Table S1**. Hazard ratios (HR) with 95% confidence intervals (CI) for all cancer and specific types of cancer, with BMI and all covariates given in the table, in patients with type 2 diabetes using the model with BMI per 5 units increase as main exposure.

|  | All cancer | Gastrointest. cancer | Colorectal cancer | Prostate cancer | Breast cancer |
| --- | --- | --- | --- | --- | --- |
|  | HR (95% CI) | HR (95% CI) | HR (95% CI) | HR (95% CI) | HR (95% CI) |
| **All patients** |  |  |  |  |  |
| BMI per 5 units | 1.08 (1.04-1.12) | 1.08 (1.01-1.17) | 1.11 (1.01-1.21) | - | - |
| Age, years | - | 1.05 (1.04-1.06) | 1.05 (1.04-1.06) | - | - |
| Diabetes duration, yrs | 0.99 (0.98-1.00) | 0.99 (0.98-1.00) | 0.99 (0.98-1.00) | - | - |
| HbA1c, % | 1.01 (0.98-1.04) | 1.03 (0.98-1.09) | 1.01 (0.94-1.07) | - | - |
| Smoking | 1.31 (1.18-1.44) | 1.28 (1.03-1.58) | 1.08 (0.83-1.41) | - | - |
| Insulin use | 1.02 (0.94-1.10) | 1.04 (0.89-1.22) | 1.10 (0.91-1.32) | - | - |
| Male sex | 0.65 (0.60-0.69) | 0.63 (0.55-0.73) | 0.67 (0.57-0.80) | - | - |
| **Men only** |  |  |  |  |  |
| BMI per 5 units | 1.05 (1.00-1.11) | 1.06 (0.95-1.18) | 1.10 (0.96-1.25) | 1.00 (0.92-1.10) | - |
| Age, years | - | 1.05 (1.04-1.06) | 1.05 (1.04-1.06) | - | - |
| Diabetes duration, yrs | 0.99 (0.99-1.00) | 1.00 (0.98-1.01) | 0.99 (0.98-1.01) | 0.99 (0.98-1.00) | - |
| HbA1c, % | 0.99 (0.96-1.02) | 0.96 (0.90-1.03) | 0.93 (0.85-1.02) | 1.00 (0.94-1.06) | - |
| Smoking | 1.30 (1.15-1.47) | 1.19 (0.92-1.54) | 1.08 (0.78-1.49) | 0.99 (0.78-1.25) | - |
| Insulin use | 0.96 (0.87-1.05) | 1.04 (0.85-1.27) | 1.01 (0.80-1.29) | 0.90 (0.76-1.07) | - |
| **Women only** |  |  |  |  |  |
| BMI per 5 units | 1.10 (1.05-1.16) | 1.10 (0.99-1.22) | 1.11 (0.99-1.26) | - | 1.14 (1.03-1.26) |
| Age, years | - | 1.04 (1.03-1.05) | 1.05 (1.03-1.06) | - | 1.02 (1.01-1.03) |
| Diabetes duration, yrs | 0.99 (0.98-1.00) | 0.99 (0.97-1.01) | 0.99 (0.97-1.01) | - | 1.00 (0.98-1.01) |
| HbA1c, % | 1.05 (1.00-1.10) | 1.14 (1.05-1.24) | 1.12 (1.01-1.23) | - | 1.01 (0.92-1.10) |
| Smoking | 1.32 (1.10-1.57) | 1.47 (1.03-2.10) | 1.10 (0.69-1.75) | - | 1.25 (0.87-1.78) |
| Insulin use | 1.13 (0.99-1.28) | 1.03 (0.80-1.34) | 1.21 (0.90-1.63) | - | 1.08 (0.83-1.39) |

Age, diabetes duration and HbA1c were continuous variables, smoking and insulin use were dichotomized variables. Stratification was performed by age quartiles for all cancer and prostate cancer.
