# Supplementary material for: Excess Body Weight and Cancer Risk in Patients with Type 2 Diabetes Who Were Registered in Swedish National Diabetes Register – Register-Based Cohort Study in Sweden
Source: PLoS One. 2014 Sep 8;9(9):e105868. doi: 10.1371/journal.pone.0105868 (PMC4157768; doi:10.1371/journal.pone.0105868)
Supplement: Table S2 — Hazard ratios (HR) with 95% confidence intervals (CI) for all cancer and specific types of cancer, with BMI and all covariates given in the table, in patients with type 2 diabetes using the model with categorized baseline BMI as main exposure. (DOCX) [file pone.0105868.s002.docx]

**Table S2**. Hazard ratios (HR) with 95% confidence intervals (CI) for all cancer and specific types of cancer, with BMI and all covariates given in the table, in patients with type 2 diabetes using the model with categorized baseline BMI as main exposure.

|  | All cancer | Gastrointest. cancer | Colorectal cancer | Prostate cancer | Breast cancer |
| --- | --- | --- | --- | --- | --- |
|  | HR (95% CI) | HR (95% CI) | HR (95% CI) | HR (95% CI) | HR (95% CI) |
| **All patients** |  |  |  |  |  |
| Overweight | 1.13 (1.03-1.23) | 1.29 (1.07-1.55) | 1.35 (1.08-1.69) | - | - |
| Obese | 1.22 (1.11-1.34) | 1.41 (1.15-1.72) | 1.52 (1.20-1.93) | - | - |
| Age, years | - | 1.05 (1.04-1.05) | 1.05 (1.04-1.06) | - | - |
| Diabetes duration, yrs | 0.99 (0.99-1.00) | 0.99 (0.98-1.01) | 0.99 (0.98-1.00) | - | - |
| HbA1c, % | 1.01 (0.98-1.04) | 1.03 (0.97-1.09) | 1.00 (0.94-1.07) | - | - |
| Smoking | 1.30 (1.18-1.44) | 1.29 (1.04-1.59) | 1.09 (0.84-1.43) | - | - |
| Insulin use | 1.01 (0.94-1.09) | 1.05 (0.90-1.23) | 1.10 (0.92-1.33) | - | - |
| Male sex | 0.65 (0.61-0.70) | 0.64 (0.55-0.74) | 0.68 (0.57-0.80) | - | - |
| **Men only** |  |  |  |  |  |
| Overweight | 1.13 (1.02-1.27) | 1.34 (1.07-1.72) | 1.59 (1.18-2.13) | 1.13 (0.94-1.36) | - |
| Obese | 1.17 (1.04-1.33) | 1.40 (1.08-1.82) | 1.62 (1.17-2.24) | 1.01 (0.81-1.25) | - |
| Age, years | - | 1.05 (1.04-1.06) | 1.05 (1.04-1.06) | - | - |
| Diabetes duration, yrs | 0.99 (0.99-1.00) | 1.00 (0.99-1.01) | 1.00 (0.98-1.01) | 0.99 (0.98-1.00) | - |
| HbA1c, % | 0.99 (0.96-1.02) | 0.96 (0.89-1.03) | 0.93 (0.85-1.01) | 1.00 (0.95-1.06) | - |
| Smoking | 1.30 (1.15-1.47) | 1.21 (0.93-1.57) | 1.10 (0.79-1.52) | 0.99 (0.79-1.25) | - |
| Insulin use | 0.96 (0.87-1.05) | 1.05 (0.86-1.27) | 1.02 (0.80-1.29) | 0.90 (0.76-1.07) | - |
| **Women only** |  |  |  |  |  |
| Overweight | 1.13 (0.97-1.32) | 1.16 (0.86-1.58) | 1.05 (0.74-1.49) | - | 0.95 (0.69-1.29) |
| Obese | 1.30 (1.12-1.51) | 1.40 (1.03-1.91) | 1.39 (0.98-1.96) | - | 1.30 (0.97-1.75) |
| Age, years | - | 1.04 (1.03-1.05) | 1.05 (1.03-1.06) | - | 1.02 (1.01-1.03) |
| Diabetes duration, yrs | 0.99 (0.98-1.00) | 0.99 (0.97-1.01) | 0.99 (0.97-1.01) | - | 1.00 (0.98-1.01) |
| HbA1c, % | 1.05 (1.00-1.10) | 1.14 (1.05-1.24) | 1.11 (1.01-1.23) | - | 1.01 (0.93-1.11) |
| Smoking | 1.31 (1.10-1.56) | 1.48 (1.03-2.11) | 1.11 (0.70-1.76) | - | 1.23 (0.86-1.75) |
| Insulin use | 1.12 (0.99-1.28) | 1.04 (0.80-1.34) | 1.21 (0.90-1.63) | - | 1.06 (0.82-1.37) |

Categories of overweight (BMI 25-29.9 kg/m^2^) and obese (BMI ≥30 kg/m^2^) were compared with normal weight as reference. Age, diabetes duration and HbA1c were continuous variables, smoking and insulin use were dichotomized variables. Stratification was performed by age quartiles for all cancer and prostate cancer.
